# Uncovering associations between data-driven learned qMRI biomarkers and chronic pain

**DOI:** 10.1038/s41598-021-01111-x

**Published:** 2021-11-09

**Authors:** Alejandro G. Morales, Jinhee J. Lee, Francesco Caliva, Claudia Iriondo, Felix Liu, Sharmila Majumdar, Valentina Pedoia

**Affiliations:** 1 Department of Bioengineering, University of California, Berkeley, USA; 2 Center for Intelligent Imaging, University of California, San Francisco, USA; 3 Department of Epidemiology and Biostatistics, University of California, San Francisco, USA

**Keywords:** Musculoskeletal system, Biomarkers, Pain

## Abstract

Knee pain is the most common and debilitating symptom of knee osteoarthritis (OA). While there is a perceived association between OA imaging biomarkers and pain, there are weak or conflicting findings for this relationship. This study uses Deep Learning (DL) models to elucidate associations between bone shape, cartilage thickness and T_2_ relaxation times extracted from Magnetic Resonance Images (MRI) and chronic knee pain. Class Activation Maps (Grad-CAM) applied on the trained chronic pain DL models are used to evaluate the locations of features associated with presence and absence of pain. For the cartilage thickness biomarker, the presence of features sensitive for pain presence were generally located in the medial side, while the features specific for pain absence were generally located in the anterior lateral side. This suggests that the association of cartilage thickness and pain varies, requiring a more personalized averaging strategy. We propose a novel DL-guided definition for cartilage thickness spatial averaging based on Grad-CAM weights. We showed a significant improvement modeling chronic knee pain with the inclusion of the novel biomarker definition: likelihood ratio test p-values of 7.01 × 10^–33^ and 1.93 × 10^–14^ for DL-guided cartilage thickness averaging for the femur and tibia, respectively, compared to the cartilage thickness compartment averaging.

## Introduction

Knee pain is the most prominent and debilitating symptom of knee osteoarthritis (OA), a degenerative joint disease which affects over 13% of U.S. adults^1^. Notably, knee pain affects up to 7.3% of the total US population over 25 years of age, and the costs of medical care and loss of productivity are rising^2^. The development of OA involves all joint tissues and is characterized by changes in the cartilage and bone. Given the lack of noninvasive treatment options to reverse the progression of structural joint degeneration, the medical care of OA has shifted to symptomatic pain management in a clinical setting^3,4^. While there is a widely perceived association of structural joint change with pain, previous studies linking OA imaging biomarkers to the presence of knee pain have not yet verified a strong correlation^5–8^.

The sources of OA-related knee pain are not yet fully understood, with tissues such as bone and cartilage implicated through direct and indirect mechanisms. In particular, the aneural nature of cartilage obfuscates its involvement in the pain process, with surrounding tissue interactions being proposed as the source of pain^9^. Structurally, OA pathogenesis is marked by progressive degradation of the cartilage extracellular matrix, with early-stage changes including cartilage hydration, proteoglycan loss, and disruption of collagen. This process can be observed using quantitative Magnetic Resonance Imaging (qMRI) through imaging biomarkers such as T_2_ relaxation time^10^. Late-stage OA is characterized by cartilage dehydration and structural breakdown, which results in measurable cartilage thickness loss on high resolution 3D MRI^11^. Alongside these cartilage changes, remodeling also occurs in the trabecular and subchondral bone, which can be observed with MRI-derived bone shape measurements^12^. Some early bony changes such as bone marrow lesions (BML) can predate cartilage degeneration, while presence of large osteophytes can act as a measure of advanced OA severity^13^.

These imaging biomarkers (cartilage T_2_, cartilage thickness and bone shape) have been classically extracted through compartment averaging, with femur, tibia, and patella divided into two or more functional regions^14,15^. This is an intuitive approach, given the prevalence of medial OA observed in patient populations, and there is particular emphasis placed in the medial compartment when conducting quantitative analysis of these biomarkers. While predictive models built with these imaging biomarker definitions tend to be interpretable, they suffer from decreased data granularity and statistical power. Furthermore, the discordance between OA-related imaging biomarkers and knee pain suggests that this methodology could be too reductive for a complex and multifactorial disease such as OA.

The advent of supervised feature learning and deep convolutional neural networks (CNN) architectures in medical image diagnostic tasks shows promising results in fully exploiting the image information by learning the most relevant data representation for the specific task considered^16–18^. However, the use of deep learning (DL) methods involve a tradeoff between model interpretability and performance, with classical rule-based expert systems^19^ and regression models being highly interpretable but not as accurate. In the last few years, a renewed focus on DL model interpretability has produced explanatory techniques such as linear proxy models, decision trees, and saliency mapping^20,21^. These approaches attempt to understand the DL model performance by approximating CNNs to linear models, decomposing CNNs into decision trees, or systematically perturbing the inputs to discover the effect on the outputs. Saliency mapping in particular, has the benefit of being scalable by directly probing the gradients in a neural network to generate visualizations of local decision-making importance for a specific input image. Among these, Gradient-weighted Class Activation Mapping (Grad-CAM) has the added benefit being class-discriminative by using the gradient information flowing into the last convolutional layer of the CNN to understand each neuron for a decision of interest^22^. The resulting class-specific saliency map can be visualized as a heat map of location importance overlaid on the input image. Grad-CAM strikes a balance between emphasizing input image regions of high network activation, where neurons fire strongest, and input image regions of high network sensitivity, where changes would most affect the decision.

This study aims to uncover latent relationships between chronic knee pain and three MRI-based OA imaging biomarkers; cartilage T_2_, cartilage thickness and bone shape by explaining CNN decisions using Grad-CAM. As a secondary aim, we propose a novel DL-guided and personalized definition of cartilage thickness compartment averaging based on Grad-CAM activations. We hypothesize these DL-guided imaging biomarkers will better explain chronic knee pain over classically extracted image biomarkers through a priori defined compartment averaging.

## Results

### Bone and cartilage segmentation

Supplementary Table S1 summarizes the performance of the bone and cartilage segmentation models using Dice Score Coefficient (DSC) and mean point to surface distance (MPTS) errors, with corresponding 95% confidence intervals (95% CI). Supplementary Figure S2 shows representative slices of the 3D bone and cartilage segmentation results from three different patients along with their respective MR images with the mean MPTS distance errors over the entire volume.

A previous study compared the fully automatic cartilage segmentation and thickness measurements with 4299 manual measurements publicly available on the OAI website. Pearson’s correlation coefficients ranged between 0.850 in central Lateral Femur (cLF) and 0.955 in Lateral Tibia (LT); average absolute difference ranged between 0.108 mm in Medial Tibia (MT) and 0.143 mm in cLF^23^. The bone segmentation was also previously extensively evaluated, with a stratified analysis showing no significant differences in segmentation performances at different KL gradings. Additionally, high performance in detecting small, relevant osteophytes was previously shown^24^.

### Spherical transformation validation

The spherical transformation method was validated over the dataset for both the average cartilage thickness and the average cartilage T_2_ time values for the femur, tibia and patella. Supplementary Figure S3 shows Bland–Altman plots comparing the original average values of cartilage thickness and cartilage T_2_ values to the spherically transformed average values for each bone. The differences between the average biomarker values were calculated using the original average values as a reference, by subtracting the original average values from the average spherical values for each biomarker. The average cartilage thickness deviations between the original and spherically transformed average data were within the in-plane pixel resolution for the 3D-DESS volumes. The slope for the spherical cartilage thickness measurements of the tibia and patella stems from the spherical transformation not preserving relative surface areas, with thicker cartilage in the central region of the tibia and patella being sampled more densely.

### Chronic pain model performance

The results of the model optimization were evaluated using the validation sensitivity, specificity, and area under the curve (AUC) as well as the coefficient of variation of the validation AUC, as a measure of training smoothness. Supplementary Figure S4 summarizes the optimization results for the best performing models for each initialization strategy. The OA pretrained Resnet50 models consistently outperformed the randomly initialized models and exhibited smoother validation AUC than the ImageNet pretrained models. The model optimization informed the global selection of a Resnet50 pretrained to predict OA and fine-tuned to predict chronic pain for all 18 models, with the individual selection of the optimal learning rate and layer freezing for each model.

The test Receiver Operating Characteristic (ROC) curve results, defined as the sensitivity, the specificity, and AUC for the binary pretraining OA diagnosis task models, along with their respective 95% CI, are summarized in Supplementary Table S2. The ROC metrics are given for each single biomarker and biomarker fusion pretraining OA diagnosis task models for each bone, as well as the ensembled averaged performance across all bones. The test sensitivity, specificity, and AUC respectively, ranged from 67.5 (95% CI 67.3, 67.7), 73.9 (95% CI 73.7, 74.1), and 77.6 (95% CI 77.5, 77.8) to 78.2 (95% CI 78.0, 78.3), 89.6 (95% CI 89.5, 89.7), and 91.7 (95% CI 91.6, 91.8). The bone shape model was the best performing single biomarker model for all bones. The femur biomarkers were the best performing models, followed by the tibia and the patella biomarker models.

For the chronic knee pain models, based on the results of the model optimization, the best model combination consisted of Resnet50 with OA pretraining, which were used for the test results. The test results included the first timepoints of each unique patient in the test set to avoid any timepoint correlation bias. The test sensitivity, specificity, and AUC respectively, ranged from 58.8 (95% CI: 58.3, 59.3), 67.4 (95% CI 67.0, 67.9), 68.1 (95% CI 67.7, 68.4) to 53.7 (95% CI 53.2, 54.1), 82.1 (95% CI 81.8, 82.4), 74.4 (95% CI 74.1, 74.8). The test performance followed a similar trend to the OA pretraining task, with the bone shape models outperforming the other single biomarker models for all bones. The performance across each bone also followed the decreasing trend of femur to tibia to patella. The cartilage T_2_ models had a more balanced performance and higher sensitivity compared to the bone shape and cartilage thickness models, which tended to be more specific to chronic pain. Most models tended to be more specific than sensitive to chronic pain, and biomarker fusion models showed increased performance compared to the single biomarker models. The full test ROC results, defined as the sensitivity, the specificity, and AUC for the binary chronic pain models, along with their respective 95% CI, are summarized in Table 1. Additionally, Supplementary Table S3 reports the performance for the last timepoints of each unique patient in the test set. The ROC metrics are given for each single biomarker and biomarker fusion chronic pain models for each bone, as well as the ensembled averaged performance across all bones.

**Table 1:** Bootstrapped (n = 100) test set chronic pain ROC performance for all six biomarker models per bone, as well as an average ensemble across all bones.

Biomarker type	Biomarker model	Test set ROC (sensitivity/specificity/AUC) (95% CI)			
		Patella	Tibia	Femur	PTF
Single	Cartilage T_2_	58.9 (58.3, 59.5)	49.1 (48.6, 49.7)	65.7 (65.3, 66.2)	62.7 (62.2, 63.2)
		75.4 (75.0, 75.8)	77.5 (77.2, 77.9)	65.1 (64.7, 65.6)	71.7 (71.4, 72.1)
		71.0 (70.5, 71.4)	68.3 (68.0, 68.7)	70.9 (70.5, 71.3)	73.7 (73.4, 74.0)
	Cartilage thickness	55.7 (55.1, 56.2)	48.1 (47.5, 48.7)	55.5 (55.0, 55.9)	54.5 (54.0, 55.0)
		72.2 (71.8, 72.6)	81.3 (81.0, 81.6)	77.2 (76.8, 77.5)	79.2 (78.8, 79.5)
		68.4 (68.1, 68.8)	70.7 (70.4, 71.1)	72.1 (71.7, 72.4)	72.8 (72.5, 73.2)
	Bone shape	53.6 (53.1, 54.1)	50.8 (50.3, 51.3)	**57.6 (57.1, 58.1)**	56.6 (56.1, 57.1)
		79.0 (78.6, 79.4)	78.8 (78.4, 79.1)	**77.7 (77.4, 78.1)**	80.0 (79.7, 80.4)
		70.6 (70.3, 71.0)	70.3 (69.9, 70.6)	**73.6 (73.3, 74.0)**	74.2 (73.9, 74.5)
Fusion	Morphological bone and cartilage fusion	**61.9 (61.5, 62.4)**	**50.7 (50.3, 51.2)**	50.8 (50.3, 51.4)	**53.7 (53.2, 54.1)**
		**69.8 (69.4, 70.2)**	**82.5 (82.2, 82.8)**	79.5 (79.1, 79.8)	**82.1 (81.8, 82.4)**
		**71.6 (71.3, 72.0)**	**72.5 (72.1, 72.8)**	71.7 (71.3, 72.0)	**74.4 (74.1, 74.8)**
	Morphological and compositional cartilage fusion	58.8 (58.3, 59.3)	43.1 (42.6, 43.6)	50.7 (50.1, 51.2)	54.4 (53.9, 54.8)
		67.4 (67.0, 67.9)	82.1 (81.8, 82.4)	81.3 (81.0, 81.7)	79.2 (78.9, 79.6)
		68.1 (67.7, 68.4)	68.9 (68.6, 69.3)	73.3 (73.0, 73.7)	73.1 (72.7, 73.4)
	All biomarkers fusion	48.8 (48.3, 49.4)	47.1 (46.6, 47.6)	52.9 (52.3, 53.5)	50.3 (49.8, 50.7)
		77.8 (77.5, 78.2)	82.1 (81.7, 82.4)	79.2 (78.8, 79.5)	82.3 (82.0, 82.6)
		69.9 (69.6, 70.3)	73.0 (72.6, 73.3)	71.6 (71.3, 72.0)	73.8 (73.4, 74.1)

Sensitivity, specificity, and AUC values are shown respectively, along with their corresponding 95% confidence intervals.

The best performances per bone and ensemble are bolded. PTF = Patella + Tibia + Femur ensemble. Result metrics are for the first timepoint for each patient.

Table 2 shows the result of the logistic regression model trained to predict chronic knee pain with radiological features such as KL grades, OARSI JSN grades for lateral and medial compartments, and the minimum medial JSW measurement. KL grades (OR 2.20; 95% CI 1.91, 2.52) and OARSI JSN grades for the lateral compartment (OR 1.24; 95% CI 1.02, 1.51) were statistically significantly associated with higher odds of chronic pain. The test sensitivity, specificity, and AUC respectively, of 0.80 (95% CI 0.77, 0.83), 0.55 (95% CI 0.50, 0.59), and 0.69 (95% CI 0.66, 0.71).

**Table 2:** Logistic regression model results for the association between chronic knee pain and radiological features including KL grades, OARSI JSN grades for lateral and medial compartments, and the minimum medial JSW measurement.

Variable	Estimates (95% CI)
Chronic pain (%)	33.1
OARSI JSN grades medial	− 0.12 (− 0.33, 0.10)
OARSI JSN grades lateral	**0.22 (0.02, 0.42)**
Quantitative JSW	− 0.05 (− 0.15, 0.05)
KL grades	**0.79 (0.64, 0.93)**
Age	− **0.04 (**− **0.05,** − **0.03)**
Female sex	0.13 (− 0.04, 0.31)
BMI	**0.05 (0.03, 0.07)**

In adjusted logistic regression analysis for ages, gender, and BMI, KL grades (OR 2.20; 95% CI 1.91, 2.52) and OARSI JSN grades for the lateral compartment (OR 1.24; 95% CI 1.02, 1.51) were statistically significantly associated with higher odds of chronic pain. The test sensitivity, specificity, and AUC respectively, of 0.80 (95% CI 0.77, 0.83), 0.55 (95% CI 0.50, 0.59), and 0.69 (95% CI 0.66, 0.71).

Bold p-values are significant (p-value < 0.05).

### Grad-CAM model interpretation for imaging biomarker discovery

From the first timepoint of each unique patient in the test set, amounting to 875 cases, a total of 87 *TP*_*Pain*_ cases and 184 *TN*_*NoPain*_ cases were selected, which consisted of the intersection of the correctly classified cases for all 18 models. This intersection, despite the reduction in number of samples, was selected over choosing different sets for each model in an attempt to perform an analysis that could provide a direct comparison between the different biomarkers. For the *TP*_*Pain*_ group, the average and standard deviation for the age and BMI was 63.8 ± 8.3 and 31.0 ± 5.1 respectively, with 33 male and 54 female patients. For the *TN*_*NoPain*_ group the average and standard deviation for the age and BMI was 60.1 ± 9.6 and 25.9 ± 4.2 respectively, with 77 male and 107 female patients. Additionally, the race distribution of the TP_*Pain*_ group consisted of 19 Black or African American patients, 67 white patients and 1 patient with unreported race, while for the TN_*NoPain*_ group, the race distribution consisted of 7 African American patients, 177 white patients.

**Figure 1:**
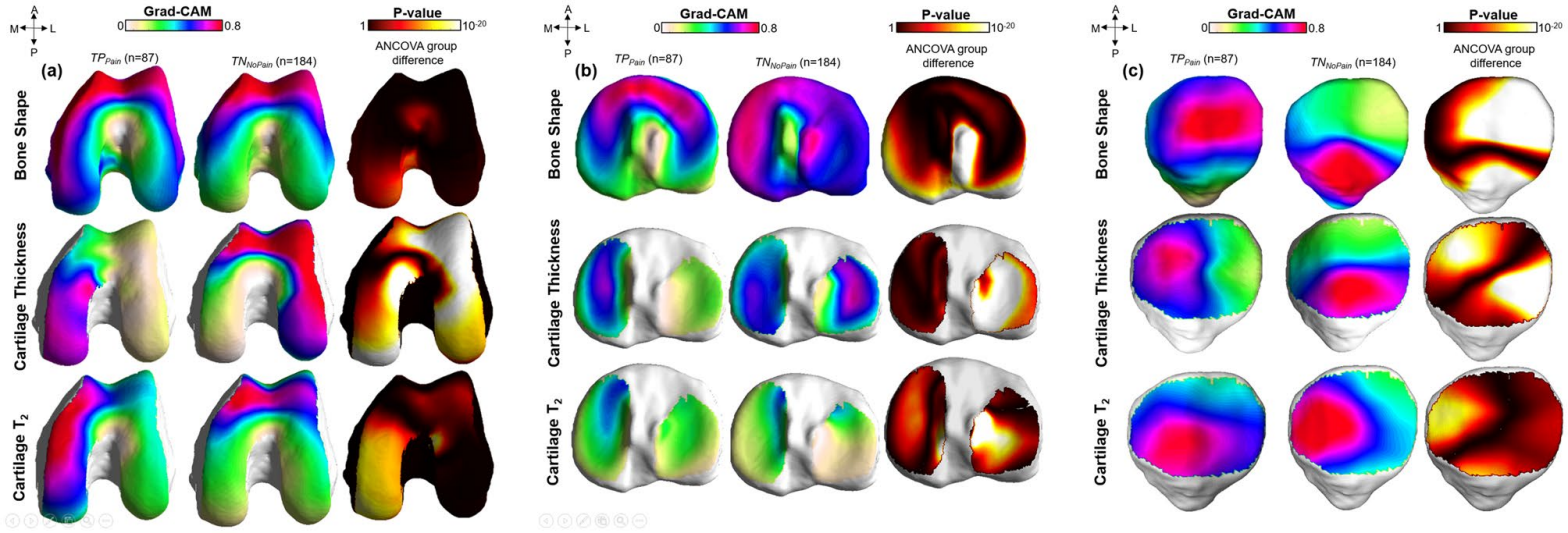
The vertices of a reference bone surface, selected to match the average demographic distribution of the test set, were mapped on all the bone surfaces in the test set using a fully automatic landmark-matching algorithm. The maximum and minimum local curvatures were used for coupling homologous points on two surfaces. Both these features were locally defined on the surfaces and used to identify the landmark matching. After the landmark matching procedure, with the heat maps in the same reference space, localized group analysis was performed to compare the true positive (*TP*_*pain*_) and true negative (*TN*_*Nopain*_) model predictions for each single biomarker. Local Statistical Parametric Mapping (SPM) was performed on these two groups to assess differences in location of important features significant for presence of pain (*TP*_*pain*_) or specific for absence of pain (*TN*_*Nopain*_). Point-by-point SPM was performed using ANOVA group comparison considering age, sex and BMI as confounding factors.

Figure 1 shows the results of the Grad-CAM statistical parametric mapping group analysis for each single biomarker for all three bones. After landmark matching, average Grad-CAM surfaces were generated for each biomarker for the two groups. The first two columns of each subfigure show the *TP*_*Pain*_ and *TN*_*NoPain*_ group average maps. In the third column, the results of the local SPM analysis are shown as a p-value surface. Figure 1a shows the results of the femur bone. For the bone shape feature, similar patterns of elevations were observed in *TP*_*Pain*_ and *TN*_*NoPain*_. In both groups, the majority of the Grad-CAM elevation was co-localized in the anterior medial femoral area. High values of these maps are indicative of common patterns in the whole group, since Grad-CAM elevations distributed in different locations for each patient would be averaged out over the group. Similar patterns in two groups, as it is observed for the femur bone shape feature, are indicative of similar location of features being exploited by the model for the assessment of both pain presence and absence.

In cartilage thickness and T_2_ imaging biomarkers, the locations of features that were sensitive for the presence of chronic pain are distinct from the locations of features that were specific for absence of chronic pain. Features sensitive for pain presence are located in the medial femoral condyle, while features that are specific for pain absence are located in the anterior femoral area, particularly in the trochlea.

Similar relationships were observed for the tibia (Fig. 1b), where the location of important bone shape features was similar in the two groups. For cartilage thickness, the medial plateau was almost exclusively observed as significant for the *TP*_*Pain*_ group while both the medial and lateral plateaus showed importance for the *TN*_*NoPain*_ group. The T_2_ biomarker in the tibia showed weak elevations in the group Grad-CAM, which demonstrates scattered peaks on the individual maps of patients.

Results on the patella bone and cartilage are shown in Fig. 1c. Bone shape biomarker features sensitive to the pain were located in the lateral facet, while features specific for absence of pain were located in the most inferior aspect of the patella bone. A similar pattern was observed for cartilage thickness, with the pattern seemingly inverted for cartilage T_2._

Table 3 shows the results of the chronic pain logistic regression using demographic factors, such as age, sex, and BMI, and the standard cartilage compartment averages compared with the same model with the addition of the DL-guided thickness averages. For the femur and tibia, the DL-guided biomarker is a significantly better predictor of the chronic pain outcome, with likelihood ratio test p-values of 7.01 × 10^–33^ and 1.93 × 10^–14^, respectively.

**Table 3:** Logistic regression results for the cartilage thickness biomarker for all bones.

Biomarker	Bone	Method	Variable	Estimate	Standard error	p-value	ROC (sensitivity/specificity/AUC)	Likelihood ratio p-value
Cartilage thickness (n = 2151)	Femur	Classical: clinical compartment average	Intercept	− 3.59	0.621	7.56 × 10^–9^	13.5 95.2 63.3	**7.01 × 10**^**–33**^
			Age	− 0.011	5.1 × 10^–3^	3.06 × 10^–2^		
			BMI	0.077	1.01 × 10^–2^	1.84 × 10^–14^		
			Sex	− 0.193	0.114	9.05 × 10^–2^		
			LF thickness	− 0.582	0.264	2.77 × 10^–2^		
			MF thickness	1.289	0.246	1.77 × 10^–7^		
		Proposed: DL-guided weighted average	Intercept	− 2.52	0.645	9.16 × 10^–5^	33.5 89.8 69.2	
			Age	− 1.97 × 10^–2^	5.36 × 10^–3^	2.33 × 10^–4^		
			BMI	5.14 × 10^–2^	1.06 × 10^–2^	1.13 × 10^–6^		
			Sex	− 8.78 × 10^–2^	0.118	0.455		
			LF thickness	2.25	0.364	6.55 × 10^–10^		
			MF thickness	2.29	0.268	1.57 × 10^–17^		
			DL-thickness	− 3.66	0.32	2.02 × 10^–30^		
	Tibia	Classical: clinical compartment average	Intercept	0.496	0.638	0.437	15.5 94.2 63.6	**1.93 × 10**^**–14**^
			Age	− 1.81 × 10^–2^	5.17 × 10^–3^	4.5 × 10^–4^		
			BMI	0.078	0.01	7.27 × 10^–15^		
			Sex	− 0.356	0.106	8.0 × 10^–4^		
			LT thickness	− 0.445	0.182	1.47 × 10^–2^		
			MT thickness	− 0.537	0.15	3.42 × 10^–4^		
		Proposed: DL-guided weighted average	Intercept	0.81	0.65	0.213	24.8 92.9 66.9	
			Age	− 2.11 × 10^–2^	5.27 × 10^–3^	6.06 × 10^–5^		
			BMI	7.03 × 10^–2^	1.02 × 10^–2^	4.86 × 10^–12^		
			Sex	− 0.387	0.107	3.12 × 10^–4^		
			LT thickness	0.289	0.208	0.165		
			MT thickness	0.108	0.173	0.533		
			DL-thickness	− 1.37	0.184	9.6 × 10^–14^		
	Patella	Classical: clinical compartment average	Intercept	1.21	0.644	6.05 × 10^–2^	15.8 94.8 65.0	0.851
			Age	− 2.38 × 10^–2^	5.33 × 10^–3^	8.09 × 10^–6^		
			BMI	6.64 × 10^–2^	1.03 × 10^–2^	1.06 × 10^–10^		
			Sex	− 0.389	0.105	2.28 × 10^–4^		
			L thickness	− 0.398	0.118	7.12 × 10^–4^		
			M thickness	− 0.424	0.12	3.97 × 10^–4^		
		Proposed: DL-guided weighted average	Intercept	1.215	0.645	5.95 × 10^–2^	16.0 94.9 65.0	
			Age	− 2.38 × 10^–2^	5.33 × 10^–3^	7.96 × 10^–6^		
			BMI	6.63 × 10^–2^	1.03 × 10^–2^	1.20 × 10^–10^		
			Sex	− 0.39	0.106	2.24 × 10^–4^		
			L thickness	− 0.376	0.166	2.39 × 10^–2^		
			M thickness	− 0.401	0.173	2.06 × 10^–2^		
			DL-thickness	− 4.57 × 10^–2^	0.243	0.851		

The demographic factors, such as age, BMI, and sex, are included to the logistic regression models as well as the different cartilage thickness averaging methods. The results are shown for the two definitions for OA imaging biomarkers, clinical compartment average and DL-guided weighted average for the femur, tibia, and patella.

*LF* Lateral Femur, *MF* Medial Femur, *MT* Medial Tibia, *LT* Lateral Tibia, *M* Medial, *L* Lateral.

## Discussion

In this study, we propose a DL-guided definition for OA quantitative imaging biomarkers which is more strongly associated to chronic knee pain than the clinical compartment average definition. We report likelihood ratio test significant p-values of 7.01 × 10^–33^ and 1.93 × 10^–14^ for DL-guided cartilage thickness averaging for the femur and tibia, respectively, compared to the cartilage thickness compartment averaging, for predicting chronic pain. The difference is reported even with the inclusion of demographic factors such as age, BMI, and sex to the regression models, which have been linked to pain^25^. This method for quantitative imaging biomarker discovery is specific to each patient, instead of being predefined based on clinical assumptions, which suggests there are personalized changes not reflected by known OA-related regions.

The average Grad-CAM saliency maps for the *TP*_*Pain*_ and *TN*_*NoPain*_ groups revealed an interesting heterogeneity in the localization of the features sensitive to pain and specific to no pain. This observation of distinct locations for pain specific and non-pain specific features for the cartilage thickness biomarker was surprising and previously unreported, to the best of our knowledge. The activation regions for the cartilage thickness across all bones showed pain specific features generally located in the medial side, while the non-pain specific features were generally located in the lateral side. This finding generated the hypothesis that the weak association between cartilage thickness and clinically relevant outcomes, such as pain, could be partly attributed to patient-specific heterogenous importance in the locations of cartilage thickness variation. Furthermore, this process might explain why the use of averages across the entire compartment would produce a weak association or even a discordance between the imaging biomarkers and pain. This selectivity between pain and non-pain specific features could be indicative of local regulatory behavior for knee pain, where areas that produce the pain could be mediated by areas associated with a lack of pain.

It is worth noting that the purpose of the study was not to achieve the highest predictive performance for chronic pain, but rather to understand local associations between the biomarkers and chronic pain. For added comparison with traditional approaches to predicting pain, we included an adjusted logistic regression model trained with radiological features. This model achieved an AUC comparable to our models and found KL grades and OARSI JSN grades for the lateral compartment to be statistically significantly associated with higher odds of chronic pain. The heterogeneity in the region importance in the SPM analysis suggests that this significance may not be reliable in singling out any compartment as the main source of pain, due to the observed differences between patients. A method which takes into account unique patient characteristics may be better suited to understand the mechanisms underlying pain at the individual level.

A recent study by Bacon et al.^26^ found a weak association between medial femorotibial cartilage thickness loss and knee pain, reporting a significant 0.32 ± 0.11 mean change in WOMAC pain scores resulting from a 0.1 mm cartilage thickness loss over a 24 month period. This correlation, while statistically significant, did not surpass the minimally clinical importance difference for WOMAC pain scores^27^. Similarly, a reduction in central medial femorotibial compartment cartilage thickness was reported to be weakly associated with pain progression with an odds ratio of 1.3 ± 0.2^28^. Our work has two key differences with these studies, the definition of chronic knee pain, instead of pain defined by the WOMAC scale, and the use of DL-guided cartilage thickness averaging, instead of compartment averaging. Our use of chronic knee pain as a clinical outcome has the advantage of focusing on persistent pain experienced over the course of a year, which is likelier to capture meaningful changes in cartilage thickness compared to the week-long recall period for WOMAC pain scores. The DL-guided approach serves as a personalized approach for region of interest definition, which allows for the extraction of an imaging biomarker more associated to pain.

The bone shape biomarker has generally been described in previous works using statistical shape modelling to compare different shape variations between case groups^29,30^. Unlike cartilage thickness and cartilage T_2_ biomarkers, there is no obvious way to apply the Grad-CAM saliencies to the bone shape maps, since averaging bone shape values may not be appropriate. For cartilage T_2_, we did not find a difference in the association between classical compartment averaging and the DL-guided weight averaging to chronic pain. While cartilage T_2_ times have been shown to be associated with pain^31^, we did not find an improvement in the inclusion of the DL-guided weight averaging to the classical compartment averaging in the regression models. This suggests that the nature of the behavior for cartilage thickness and cartilage T_2_ may be different, with the latter exhibiting a weaker pain feature heterogeneity. Compartment averaging for T_2_ relaxation times may be sufficient in explaining chronic pain.

Although this study brings new insights on the role of deep learning for quantitative imaging biomarker discovery, several limitations need to be acknowledged. One of the limitations of the study is the focus on structural changes, which omits the impact of inflammatory changes that have been consistently linked to pain. Bone marrow lesions and synovitis, in particular, have been reported to play a role in the pain process and are not directly reflected by our biomarkers^32^. Additionally, the pain performance improvement of the biomarker fusion models over the single biomarker models suggests that there are some added pain-related interactions between biomarkers. These were not further inspected due to the reduced interpretability of combining the biomarkers at the input level. The use of the intersection of all 18 models limited the findings to the set of imaging features that are most persistently associated with chronic pain. This could result in the loss of more nuanced patient-specific relationships to pain. The definition of chronic knee pain only takes into consideration the presence of pain but not the severity of the pain. The OAI is also a limited dataset and findings based on it may not be generalizable to the general population. Another limitation of the OAI is the presence of MRI artifacts due to patient motion, magic angle effect, chemical shift, and fluid from bone marrow lesions, which may limit the accuracy of the tissue segmentations and the T_2_ relaxation time values.

The findings of this work could improve the imaging biomarker definition for clinical trials, with patient-specific imaging biomarkers that are more strongly correlated to clinical outcomes such as pain. A recent clinical trial for the disease-modifying osteoarthritis drug sprifermin showed a protective effect for femorotibial average cartilage thickness loss of 0.1 mm over a period of 2 years^33^. The same trial found no significant effect for this substantial cartilage preservation on the WOMAC pain scores, which highlights the importance of stronger predictors for pain. Our proposed DL-guided cartilage thickness averaging could be used to evaluate the effect of such cartilage-preserving treatments on pain, tailoring the imaging biomarker to the clinical outcome.

## Methods

### Aim and study overview

This study uses three known OA quantitative MR imaging biomarkers: bone shape, cartilage thickness and T_2_ relaxation times, to train OA-related chronic knee pain classification models. It then leverages the trained models to determine the spatial averaging weights for each biomarker that are most correlated to chronic knee pain classification. In the next paragraph we present an overall study overview, with all the steps explained in detail in the subsequent sections.

First, the biomarkers are extracted from the knee MRI dataset by using two automatic segmentation models for the femur, tibia, and patella bones and corresponding cartilage. The cartilage thickness and T_2_ relaxation times are then calculated from the cartilage segmentations while the bone shape is calculated from the bone segmentations. The three biomarkers are projected into the surface of the femur, tibia, and patella bones and transformed into spherical coordinates to obtain 2D images. Six different strategies were performed to merge biomarker spherical maps for each bone. Each of the six strategies for each bone was used to train individual chronic knee pain classification models, which were pretrained to classify radiographic OA, for a total of 18 models. Grad-CAM interpretation spherical maps of the entire hold out test set for all chronic knee pain models were inverted to the original bone surfaces and harmonized to a single atlas. Local group analysis of the two true predictive groups, true positives and true negatives, were compared to assess the local spatial difference in pain features for each group using a statistical parametric mapping technique. Two cartilage thickness averages were obtained using classically identified clinical compartments and using the Grad-CAM for each patient as a local weighting factor of the averaging (DL-guided). Logistic regression models were then used to compare the associations of DL-guided OA quantitative imaging biomarkers and a priori clinical compartments average biomarkers to chronic knee pain.

### Imaging dataset

The imaging data for this study was acquired from the Osteoarthritis Initiative Dataset (OAI), a multi-center longitudinal multimodality imaging study in 4796 patients^34^. This dataset consisted of a total of 12 time points ranging from an initial baseline visit to a final 108 months visit with yearly visits in between and a half-year visit for the third and fifth visits. Demographic data such as age, body mass index (BMI), and sex, was recorded during each visit. Additional details of data collection and study design have been previously reported^34^. The OAI study protocol was approved by the National Institute of Arthritis and Musculoskeletal and Skin Diseases (NIAMS) and is registered on ClinicalTrials.gov as “Osteoarthritis Initiative (OAI): A Knee Health Study”, NCT#00080171. The study was carried out in accordance with all pertinent guidelines and regulations, and written and informed consent was obtained from participants prior to each clinical visit in the study.

Two MRI sequences from the OAI were evaluated in this study, 3D Sagittal Double Echo Steady-State (3D-DESS) structural sequence and a 2D Sagittal Multi-Slice Multi-Echo (2D-MSME) spin-echo T_2_ compositional sequence.

### Clinical outcome definition

Chronic pain labels were defined using clinical data from the OAI available for a subset of the patients. The chronic pain label was defined as patient timepoints which reported a knee pain, aching, or stiffness more than half of the days of a month for more than 6 months of the past 12 months. The no chronic pain label was defined as patient timepoints which did not report any knee pain, aching, or stiffness in the past 12 months. To control for nonspecific sources of pain outside of the knee, we excluded patients showing the presence of wide-spread pain syndrome, defined as reported pain concurrently in above-waist joints (shoulder, elbow, wrist, hand), below-waist joints (hip, knee, ankle, and foot), and axial joints (back and neck) for more than half of the days in the previous 30 days^7^. This localized definition of chronic pain focuses on pain symptoms lasting for months compared to shorter term clinical pain definitions such as the Western Ontario and McMaster Universities Osteoarthritis Index^35^ (WOMAC) scores and the Knee injury and Osteoarthritis Outcome Score^36^ (KOOS), which focus on the previous 7 days. OA and its detectable imaging features may be more likely in patients who consistently reported pain within a yearlong period^7,37^.

### Patient inclusion

**Figure 2:**
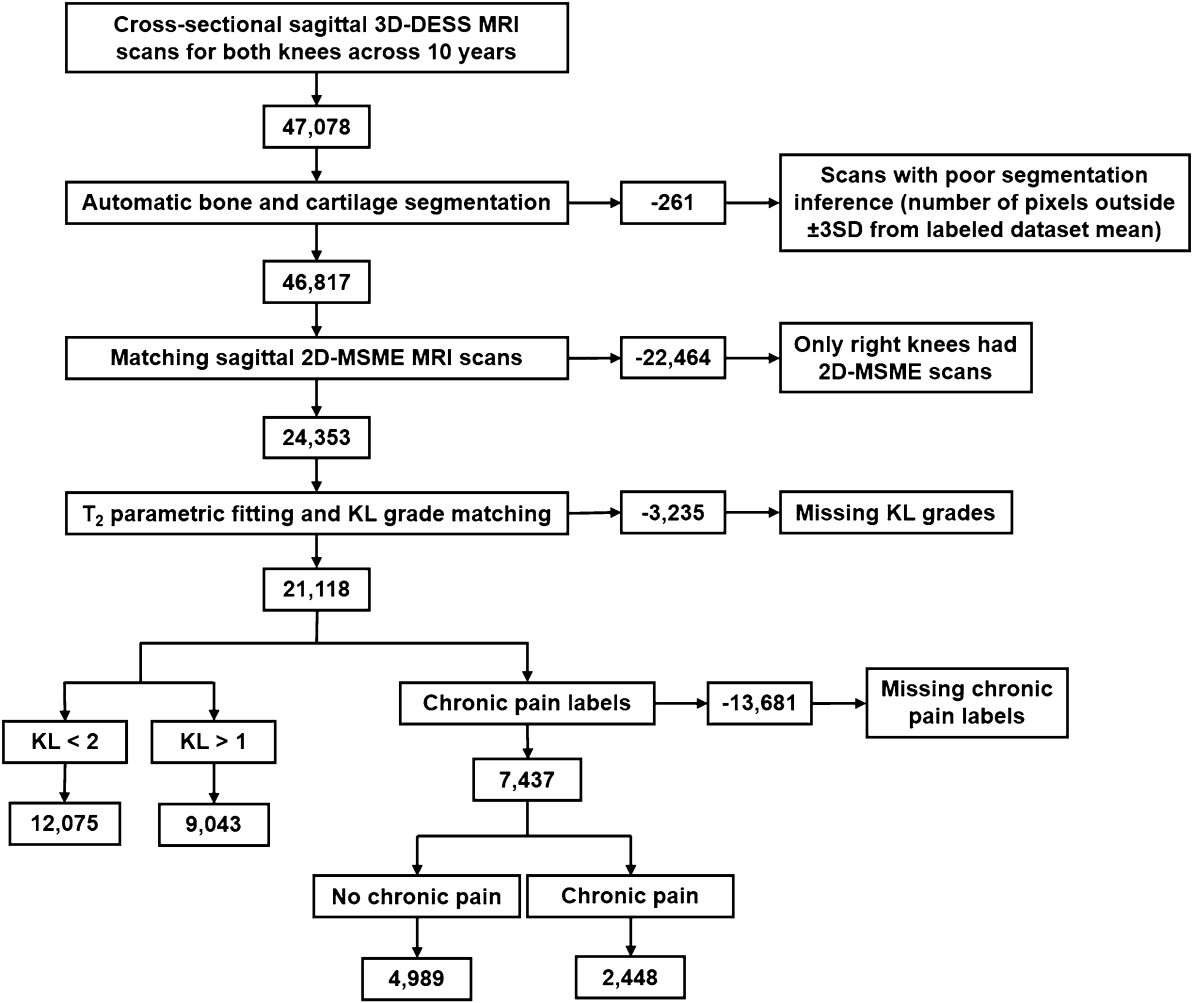
The inclusion criteria for a knee image volume from a specific patient timepoint in this cross-sectional study. The three main criteria were the existence of a KL grade, a chronic pain label, and matching 3D-DESS and 2D-MSME image volumes, which resulted in 7437 cross-sectional timepoints from 3067 unique patients.

The three main criteria for inclusion of a knee image volume from a specific patient timepoint in this cross-sectional study were the existence of a KL grade, a chronic pain label, and matching 3D-DESS and 2D-MSME image volumes. Starting with a total of 47,078 3D-DESS image volumes, 261 image volumes were excluded due to poor inference quality from the bone and cartilage segmentation models (defined as a segmentation volume outside of three standard deviations from the mean training segmentation), 22,464 image volumes from left patient knees were excluded due to absence of 2D-MSME for left knee image volumes, 3235 image volumes were excluded due to missing KL grades for the visit, and 13,681 image volumes were excluded following exclusion criteria of the chronic pain definition described above. This selection resulted in 7437 cross-sectional timepoints from 3067 unique patients. The patient selection flowchart is summarized in Fig. 2.

### Bone and cartilage segmentation

The first step of the study was to accurately segment the bone and cartilage from the 3D-DESS volumes in the OAI dataset. An ensemble of five 3D V-Net^38^ architectures, each trained with different distance-weighted loss functions^39^, was used for the femur, tibia and patella bone segmentation (Supplementary Fig. S1). A full description of the bone segmentation models can be found in Supplementary Information: Bone Segmentation.

For the cartilage segmentation, an ensemble of three 2D V-Nets and three 3D V-Nets were trained to segment femoral, tibial, and patellar cartilage and menisci (Supplementary Fig. S1). A full description of the cartilage segmentation models can be found in Supplementary Information: Cartilage Segmentation. This model was extensively validated in a previous study^23^.

### Morphometry

The cartilage thickness was calculated for each of the three cartilage masks per sagittal slice using an Euclidean distance transform along the morphological skeleton of each mask^23^. The morphological skeleton was defined as the middle points along the length of each cartilage mask. The distance transform provided the distance from each skeleton point to the edge of the cartilage, which was doubled to obtain the cartilage thickness. For full details of this automatic cartilage thickness method, we refer to a previous study^23^. The bone shape was intrinsically described by the distance from the bone surface of each bone mask to its volumetric centroid^24^.

### Relaxometry

In order to colocalize the three imaging biomarkers considered for this study; the 2D-MSME image volumes were rigidly aligned to the 3D-DESS volumes using the Patient Coordinate System (PCS) in the DICOM metadata of both MRI scans. The sagittal in-plane and coronal slice resolution of the 2D-MSME volumes were first matched to the 3D-DESS volumes using bicubic interpolation. The alignment was performed using the first echo volume, and the resulting transformation was applied to all echoes. Once the resolutions were matched, the 2D-MSME sagittal slices were spatially shifted to match the 3D-DESS sagittal slices to create MSME-DESS registered volumes. The automatically segmented cartilage mask from the 3D-DESS cartilage segmentation model was then used to isolate the cartilage from the MSME-DESS. The cartilage T_2_ relaxation time values were computed on the masked MSME-DESS echoes using a three-parameter, Levenberge-Marquardt mono-exponential: (S(TE) α exp(− TSL/T_2_) + C).

### Bone surface projection

**Figure 3:**
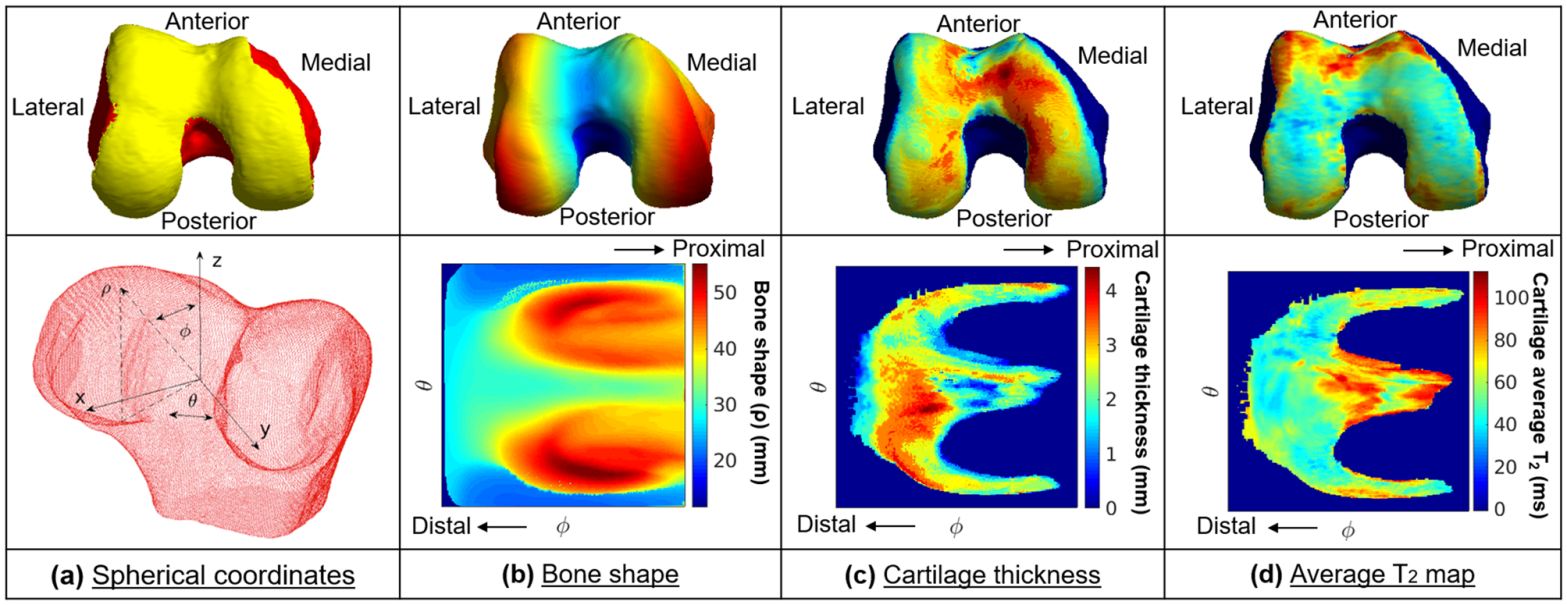
Biomarker 2D spherical maps. The three biomarkers projected to the articular bone surface were converted to 2D spherical maps. **(a)** The transformation from Cartesian coordinates into spherical coordinates was performed by uniformly sampling 224 × 224 points in the point cloud and describing them based on the angle along the x–y plane from the positive x-axis (θ), the elevation angle from the x–y plane (φ) and the distance from the center of the point cloud to the sampled point in the surface (ρ). The angle θ was sampled from − π to + π for all bones while the angle φ was sampled from − π/2 to + π/8 for the femur and tibia and from − π/2 to + π/8 for the patella. The sampling was designed to be centered around the articular surface to ensure the cartilage would be centered for each bone. **(b)** Bone shape 2D spherical map. **(c)** Cartilage thickness 2D spherical map. **(d)** Cartilage average T_2_ value 2D spherical map.

The shafts of the tibia and femur bone masks were cropped to the mediolateral length of each bone, thus creating a cubic bounding box, in order to be invariant to the different shaft lengths. The bone and cartilage masks were then converted from voxel masks to a triangulated mesh using marching cubes algorithm implemented in MATLAB, and each 3D biomarker map within the cartilage volume was then projected onto the articular bone surface (Fig. 3a). This step mapped each point in the articular surface to a value from each of the three biomarkers. The bone shape was defined as the distance from the centroid of the bone point cloud to the bone surface (Fig. 3b). The calculated cartilage thickness of the overlying cartilage was projected to each perpendicular point in the articular bone surface (Fig. 3c). The superficial, deep, and total average T_2_ values for the corresponding section of the cartilage used during the thickness projection were projected to each perpendicular point in the articular bone surface. The superficial and deep subdivisions of the cartilage used for the T_2_ averaging were defined as the respective top and bottom halves of the cartilage, with Fig. 3d showing the total average T_2_ value projection. The projection from the cartilage to the bone surface was calculated using the intersection between the normal vector for each point in the bone surface and the cartilage maps. This normal vector spanning from each point in the bone surface formed a cylinder with a radius of 0.729 mm, empirically set to double the in-plane pixel resolution, that averaged the cartilage thickness and cartilage T_2_ values along the cartilage cross-section it covered.

### Spherical transformation

In order to obtain a two-dimensional co-localized representation of the three biomarkers, cartilage thickness and cartilage T_2_ were projected to the articular bone surface and they were converted to 2D spherical maps. The transformation from Cartesian coordinates into spherical coordinates was performed by uniformly sampling 224 × 224 points in the mesh, to conform to the ImageNet^40^ image size for pretraining, and describing them based on the angle along the x–y plane from the positive x-axis (θ), the elevation angle from the x–y plane (φ) and the distance from the center of the mesh to the sampled point in the surface (ρ) (Fig. 3a). The angle θ was sampled from − π to + π for all bones while the angle φ was sampled from − π/2 to + π/8 for the femur and tibia and from − π/2 to + π/8 for the patella. Bicubic interpolation was performed between the sampled points to create densely sampled spherical maps. The sampling was designed to be centered around the articular surface to ensure the cartilage would be centered for each bone (Fig. 3b–d).

**Figure 4:**
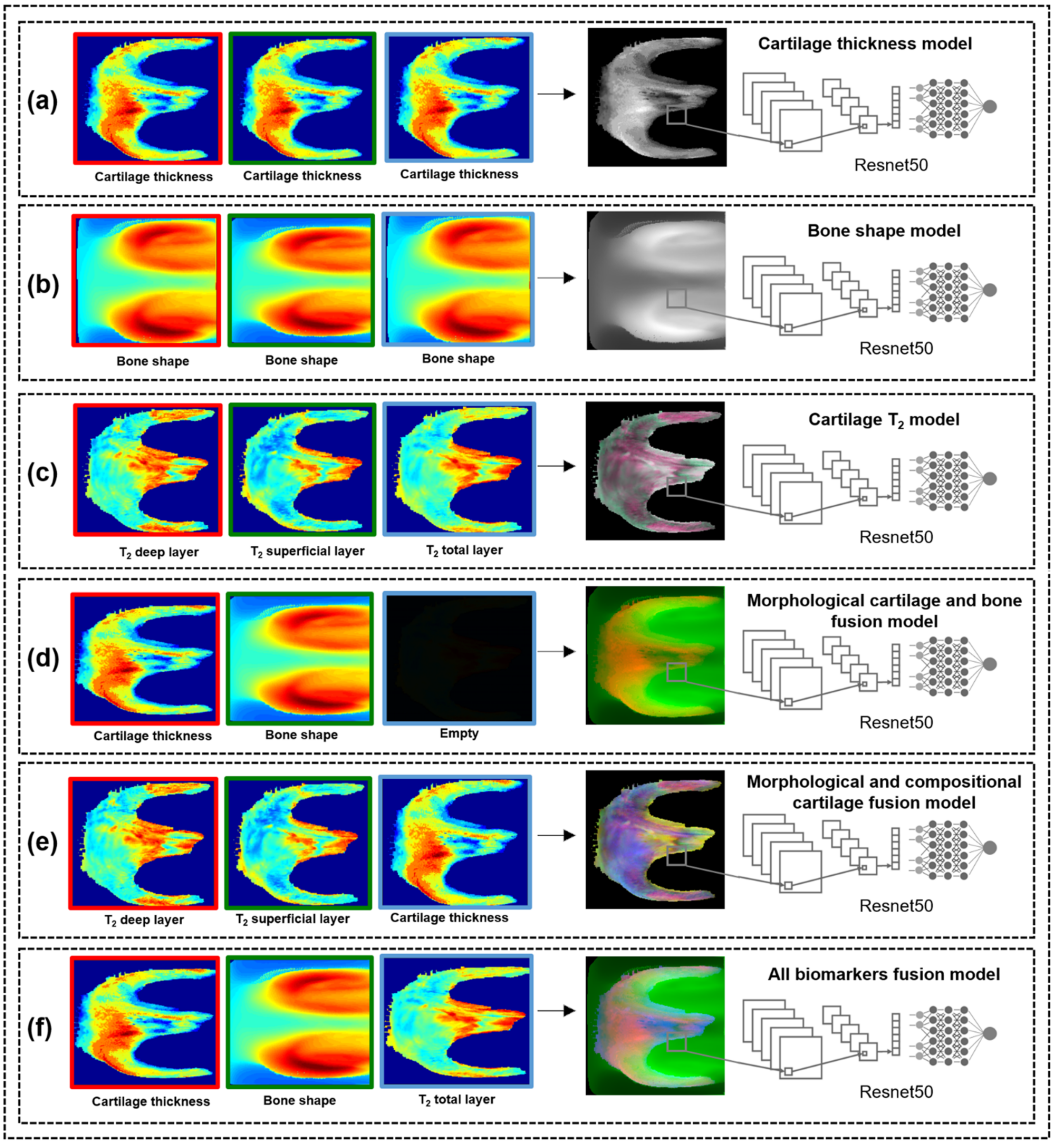
Overview of the biomarker model strategies, shown for the femur. The normalized spherical images for each patient were merged into a three-channel 8-bit image. **(a–c)** The first three strategies consisted of the single biomarkers: cartilage thickness, bone shape, and cartilage T_2_. **(a)** The cartilage thickness strategy consisted of the cartilage thickness spherical maps replicated three times into a spherical image. **(b)** The bone shape strategy consisted of the bone shape spherical maps replicated three times into a spherical image. **(c)** The cartilage T_2_ strategy consisted of the deep, superficial, and average T_2_ spherical maps as the first, second, and third channels respectively. **(d–f)** The last three fusion strategies consisted of the biomarker fusions: morphological cartilage and bone fusion, morphological and compositional cartilage fusion and all biomarkers fusion. **(d)** The morphological cartilage and bone fusion consisted of the cartilage thickness and bone shape spherical maps as the first and second channels respectively, with the last channel empty. **(e)** The morphological and compositional cartilage fusion consisted of the deep and superficial T_2_ spherical maps as the first and second channels respectively with the third channel consisting of the cartilage thickness spherical map. **(f)** The all biomarkers fusion consisted of the cartilage thickness, bone shape, and average T_2_ spherical map as the first, second and third channels respectively.

The spherical images were group normalized by the minimum and maximum biomarker value from each bone for all the patients. The normalized spherical images for each patient were merged into three-channel 8-bit images, with the six strategies shown for the femur in Fig. 4. The spherical maps were directly colocalized for each bone, with each point describing the same geometric location in the articular surface. This colocalization allowed the model to learn local features that arise from interactions between the different biomarkers across the same bone. Each channel was normalized separately. To illustrate for the morphological and compositional cartilage fusion (Fig. 4e), a pixel in the spherical image with elevated T_2_ values for both the deep and superficial cartilage layers as well as cartilage thinning could have a 3-channel value of (204, 204, 26), which would be a dark yellow. Another pixel in the same spherical image with elevated T_2_ values for the superficial cartilage layer with average cartilage thickness and T_2_ values for the superficial cartilage layer could have a 3-channel value of (128, 204, 128), which would be a dark green.

### Chronic pain model training

A total of 18 binary classification models, one for each biomarker strategy per bone, were trained to extract biomarker features from the spherical biomarker representations and use them to predict chronic knee pain (Supplementary Fig. S1). Each chronic pain model was trained using 7437 spherical images divided into 4029 training images, 1257 validation images and 2151 test images, with no patient overlap across splits. To test the independence of demographic factors (sex, age, BMI) for the chronic pain cases across splits, two different statistical tests were performed. The independence of sex was tested with a Pearson’s chi-squared test while the independence of age and BMI was tested with a one-way MANOVA. Table 4 summarizes the training, validation and test set splits for the segmentation and classification models, along with the p-values of the statistical tests showing independence of demographic factors.

**Table 4:** Training, validation, and test splits information for the segmentation and classification models.

Task	Model	Training (cases)	Validation (cases)	Test (cases)	Cases ratio	Average time points per patient	Timepoint distribution (number of timepoints per number of patients)	Average age (mean ± SD)	Average KL (mean ± SD)	Sex distribution (male/female)	Total WOMAC pain scores (mean ± SD)	χ^2^ test correlation (sex) (p-values)	MANOVA one-way correlation (age|BMI) (p-values)
Segmentation	Bone	57 (29)	15 (8)	30 (16)	0.520	1.01	1:100	58.4 ± 8.19	0.6 ± 1.06	49/53	2.4 ± 2.90	0.745	0.413
							2:1						
	Cartilage	118 (114)	28 (28)	28 (28)	0.977	2.0	2:87	61.6 ± 9.93	2.3 ± 0.94	90/84	4.3 ± 3.80	0.156	**1 × 10**^**–4**^
Classification	OA	12,634 (5402)	2558 (1111)	5926 (2530)	0.428	4.78	1:179	63.2 ± 9.17	1.3 ± 1.21	9005/12,113	2.1 ± 2.95	0.121	0.190
							2:396						
							3:419						
							4:601						
							5:1367						
							6:527						
							7:927						
	Chronic pain	4029 (1324)	1257 (411)	2151 (713)	0.329	2.42	1:1103	63.9 ± 9.38	1.2 ± 1.22	3510/3927	1.5 ± 2.77	0.179	0.0848
							2:771						
							3:509						
							4:345						
							5:192						
							6:104						
							7:43						

Demographic factors were controlled by testing for statistical independence across the splits using a Pearson’s chi-squared test (χ^2^) for the categorical sex variable and a one-way Multivariate Analysis of Variance (MANOVA) for the joint effect of age and BMI.

Bold p-values are significant (p-value < 0.05).

The chronic pain prediction models were pretrained on an OA classification task. There were 21,118 cross-sectional timepoints from 4,416 unique patients. The KL grade distribution consisted of 8103 (KL = 0), 3972 (KL = 1), 5335 (KL = 2), 2897 (KL = 3) and 811 (KL = 4). The KL grades represent no OA (KL = 0), minimal/doubtful OA (KL = 1), mild OA (KL = 2), moderate OA (KL = 3), and severe OA (KL = 4). For the purposes of this study, KL grades of 0 and 1 were determined to be healthy while KL grades of 2, 3, and 4 are considered to be OA.

This study evaluated three types of Resnet^41^ architectures with 18, 34, and 50 layers (Resnet18, Resnet34, Resnet50) with a binary class output. The Resnet18 and Resnet34 architecture consists of stacked building blocks of two convolutional layers with a 3 × 3 convolutional filter size, while the Resnet50 architecture follows the pattern of three convolutional layers with a 1 × 1, 3 × 3, and a 1 × 1 convolutional filter size respectively. For all architectures, each convolutional layer is paired with batch normalization and a rectified linear unit activation function.

Model training optimization for all 18 models was performed using the training and validation splits with two different learning rates (1 × 10^–4^ and 1 × 10^–5^), three types of Resnet (Resnet18, Resnet34, Resnet50), three initialization strategies (Random^42^, ImageNet, OA), and four variants of layer freezing during training (first layer, first two layers, all layers, no layers), for a total of 612 combinations. The model optimization was performed with Adam optimizer for 100 epochs with an early stopping 15-epoch patience for validation loss non-improvement over the best validation loss reached. The models were trained end to end using a class-weighted binary cross entropy loss, based on the class imbalance, with a batch size of 300. The test set was held out for each model during training optimization and the test performance was evaluated just once for the optimal 18 models.

As a comparison of our models to traditional methods, a logistic regression model adjusted for age, sex, and BMI measured the association between chronic knee pain and radiological features such as KL grades, Osteoarthritis Research Society International (OARSI) Joint Space Narrowing (JSN) grades for lateral and medial compartments, and the minimum medial Joint Space Width (JSW) measurement. This model was trained using the same splits as the 18 models.

### Grad-CAM model interpretation for imaging biomarker discovery

The overreaching goal of this study is to uncover associations between quantitative MR imaging biomarkers and chronic knee pain. We used the Grad-CAM model interpretation technique to obtain a class discriminative localization map for each prediction. We first compute the gradient of the class of interest (before the softmax function) with respect to feature maps of the last convolutional layer in the Resnet. These gradients flowing back are global average-pooled to obtain the neuron importance weights for the target class. A heat map of location importance is then up sampled to match the image size and overlaid on the input image.

**Figure 5:**
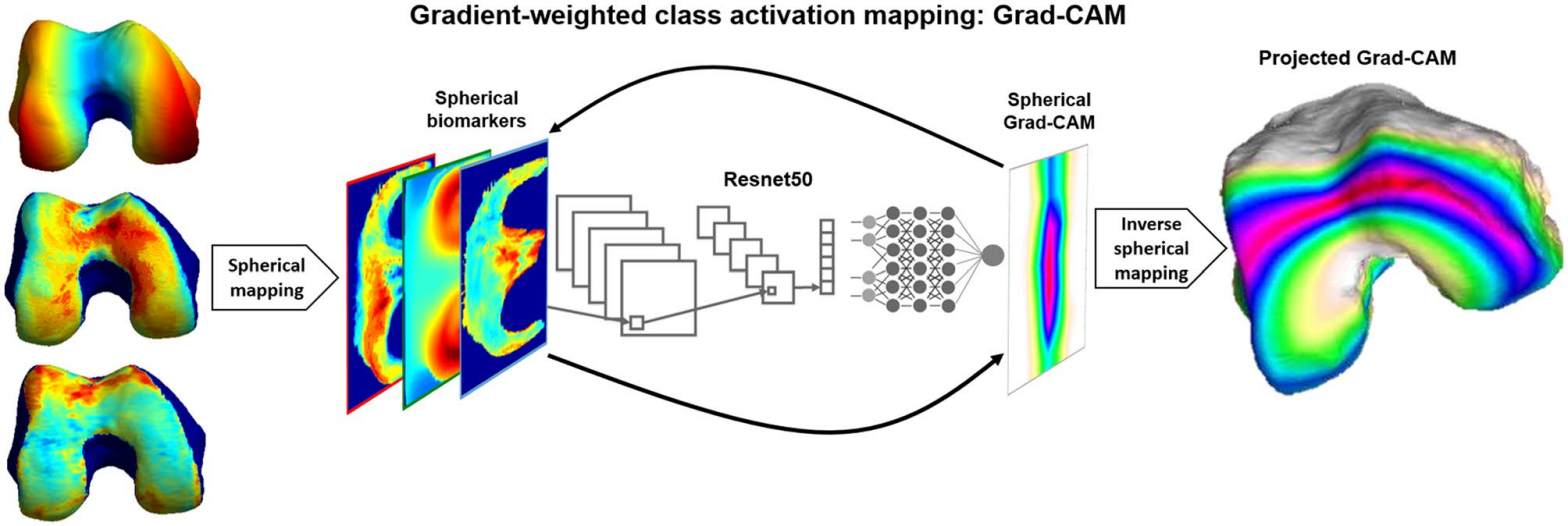
We used the Grad-CAM model interpretation technique to obtain a class discriminative localization map for each prediction. We first computed the gradient of the class of interest (before the softmax function) with respect to feature maps of the last convolutional layer in the Resnet. These gradients flowing back are global average-pooled to obtain the neuron importance weights for the target class. A heat map of location importance is then up sampled to match the image size and overlaid on the input image. We then leveraged the invertible property of our spherical transformation method to generated articular surface importance heat maps for model interpretation for each bone and for each single biomarker. This process was performed on the first timepoint of every unique patient in the hold out test set (n = 875) and is illustrated for the femur.

We leveraged the invertible property of our spherical transformation method to generate articular surface importance heat maps for model interpretation for each bone and for each single biomarker. This process was performed on the first timepoint of every unique patient in the hold out test set (n = 875) and is illustrated for the femur on Fig. 5.

The vertices of a reference bone surface, selected to match the average demographic distribution of the test set, were mapped on all the bone surfaces in the test set using a fully automatic landmark-matching algorithm. The strategy used in this study was based on the one proposed by Lombaert et al.^43^. The maximum and minimum local curvatures were used for coupling homologous points on two surfaces. Both these features were locally defined on the surfaces and used to identify the landmark matching solved using Coherent Point Drift^44^. After the landmark matching procedure, with the heat maps in the same reference space, localized group analysis was performed to compare the true positive (*TP*_*Pain*_) and true negative (*TN*_*NoPain*_) model predictions for each single biomarker. Local Statistical Parametric Mapping (SPM) was performed on these two groups to assess differences in location of important features significant for presence of pain (*TP*_*Pain*_) or specific for absence of pain (*TN*_*NoPain*_). Point-by-point SPM was performed using ANOVA group comparison considering age, sex and BMI as confounding factors.

An ad-hoc analysis was then performed to compare the ability to explain chronic knee pain between cartilage thickness imaging biomarkers averaged using clinical compartments and a novel DL-guided definition based on weight averaging of the cartilage thickness with the scaled values of Grad-CAM as weights. Two logistic regression models were built to predict chronic knee pain, both with age, BMI, sex, and clinical compartment cartilage thickness averages, and one with DL-guided cartilage thickness averages. The performance of the nested models was compared using a likelihood ratio χ^2^ test to determine the significance of the improvement of adding the DL-guided cartilage thickness averages. The linearity of the regression models and simplification of the analysis was used to compare the associations with pain of the classical and DL-guided biomarkers, instead of identifying nonlinear associations between the biomarkers and pain.

## Supplementary Information

Supplementary Information.

## Data Availability

The datasets analyzed during the current study are available through the Osteoarthritis Initiative, which can be accessed at https://nda.nih.gov/oai/. In addition, model checkpoints, code, and label files used to produce presented results can be accessed at https://github.com/alemorm/deep-pain.
