# Supplementary material for: Uncovering associations between data-driven learned qMRI biomarkers and chronic pain
Source: Sci Rep. 2021 Nov 9;11:21989. doi: 10.1038/s41598-021-01111-x (PMC8578418; doi:10.1038/s41598-021-01111-x)
Supplement: Supplementary file 1 — Supplementary Information. [file 41598_2021_1111_MOESM1_ESM.pdf]

Uncovering Associations Between Data-Driven Learned qMRI Biomarkers and Chronic Pain

Alejandro G. Morales[12*], Jinhee J. Lee[2], Francesco Caliva[2], Claudia Iriondo[12], Felix Liu[3], Sharmila Majumdar[2], Valentina Pedoia[2]
[1]*Department of Bioengineering, University of California, Berkeley, USA*
[2]*Center for Intelligent Imaging, University of California, San Francisco, USA*
[3]*Department of Epidemiology and Biostatistics, University of California, San Francisco, USA*
**Correspondence should be addressed to A.M. (email: alegmoralesm@gmail.com)*

BONE SEGMENTATION:

Bone segmentation network implementation:

The first step of the study was to accurately segment the bones from the 3D-DESS volumes in the OAI dataset. An ensemble of five 3D V-Net[1] architectures were trained and tested on 72 and 30 3D-DESS volumes, respectively, and used to segment the bone from the entire OAI dataset (**Supplemental Figure 1a**).

A modified V-Net architecture was adapted from an existing TensorFlow 1.0 (Google, Mountain View, CA) implementation (https://github.com/MiguelMonteiro/VNet-Tensorflow) for the femur, tibia and patella bone segmentation. The 3D V-Net architecture consisted of an encoder-decoder network with the encoder network compressing the most relevant features for the segmentation task while the decoder network decompresses these features to reconstruct the labeled segmented volume. The decoder network had five levels, with each level doubling the number of convolutional filters and using short shortcut connections between each layer input and output in the form of element-wise addition. The network also used long shortcut connections between each mirroring level by concatenating the layer output of each encoder layer to the layer input of its corresponding mirrored decoder layer. These connections have been shown to improve the uniform update of weights for deeper CNNs and improve gradient stability[2]. The activation function used after each convolution was a rectified linear unit (ReLU), trained on the last dimension of the input, and the last fully connected layer was activated with a softmax function for all the classes (femur, tibia, patella, background). Additionally, a dropout rate of 0.05 was used to improve generalizability of the model during training, randomly turning off activations at a rate of 5%.

Each of the five V-Net models was trained with a different distance-weighted loss functions[3]. The distance weighting was an added penalty to ensure that the segmentation accuracy was prioritized along the surface of the bone and cartilage. This ensured that the articular bone surface was as accurate as possible prior to the biomarker projection. Additionally, given the class imbalance between the different bones, with the femur being much larger than the patella, class weights were added to four of the losses to ensure that the learning process was balanced. The distance-weighted loss functions were: class-weighted Dice Score Coefficient (DSC) loss, class-weighted cross-entropy loss, mixed weighted cross-entropy and class-weighted DSC loss (with the weighting factor for the cross-entropy loss equal to 0.1), class-weighted penalized confident output cross-entropy loss[4], and regular DSC loss.

Bone segmentation network training:

A batch size of one sample per feed-forward was used, which was the memory limit of the graphical processing unit (GPU). The network was trained using Adam optimizer[5] with a learning rate of 5e-4 using TensorFlow 1.10 in a Titan 1080 Ti 12GB GPU (NVIDIA, Santa Clara, CA). All the weights for the 3D convolutional layers were randomly initialized with a Xavier uniform distribution[6]. The training was performed for a total of 500 epochs and stopped early after a 30-epoch patience for validation loss non-improvement over the best validation loss reached. Data augmentation was performed online with an independent 50% chance of flipping the input volume along the lateral-medial dimension and an independent 50% chance to randomly rotate the sagittal plane in a range of -5 to +5 degrees in 1-degree increments. The labels were truncated to the integer part after the 2D sagittal affine rotation to ensure there were no artificial partial volume effects introduced by the augmentation.

The bone segmentation training set consisted of 102 3D-DESS volumes that were carefully annotated by trained users. The age and BMI for the training split with the respective standard deviation was 57.2 ± 7.4 year and 27.5 ± 5.2 kg/m^2 respectively. The age and BMI for the validation split with the respective standard deviation was 60.9 ± 10.6 year and 28.9 ± 4.2 kg/m^2 respectively. The age and BMI for the test split with the respective standard deviation was 59.4 ± 7.6 year and 27.2 ± 4.7 kg/m^2 respectively. The sex split for training, validation and test splits was 31 males/26 females, 7 males/8 females, and 11 males/19 females respectively. The network training was performed with 72 patients, 57 used for training, 15 for validation. The model was evaluated using a test set with 30 unseen patient volumes. **Table 1** summarizes the distribution of OA cases and healthy controls for the bone segmentation dataset as well as the statistical independence tests for confounding demographic factors across splits.

Bone segmentation inference and ensembling

The trained V-Net bone ensemble segmentation model was used to segment the femur, tibia, and patella from a total of 47,078 3D-DESS volumes in the OAI. The inference was performed in 8 batches of 6,000 volumes and each batch lasted 3 hours. The inferred bone segmentation masks for all five models were then subsequently ensembled by averaging the softmax values for each bone across all models. The accuracy of the bone segmentation models was calculated using the DSC, which is proportional to the intersection over the union and calculates the doubled proportion of correctly classified pixels divided by the total number of pixels segmented and in the ground truth. The MPTS distance error calculated the mean of the errors between each closest points in two 3D surfaces. For large volume 3D surfaces, the MPTS becomes more useful for determining the accuracy of the segmentation on the surface, since the DSC would be skewed by the inner pixels of the volume.

CARTILAGE SEGMENTATION:

Cartilage segmentation networks implementation:

A cartilage and menisci segmentation model ensemble was trained on 148 3D-DESS volumes and tested on 28 3D-DESS volumes[7]. The trained ensemble consisted of three 2D V-Net and three 3D V-Net architectures and was used to segment the cartilage and menisci in the OAI dataset (**Supplemental Figure 1a**).

The same 3D V-Net architecture as the bone segmentation V-Net was implemented in Tensorflow 1.10. The 2D V-Net architectures were derived from the 3D V-Net, where the convolution kernels are modified to accommodate 2D data. The 2D V-Nets were 2 levels deep with 4 convolutions at each level, and 4 convolutions at the bottom level, all activated with ReLU functions. At the output layers, a softmax activation produced the tissue segmentations. Dropout was used to improve generalizability of the model during training, randomly turning off activations at a rate of 5%.

Cartilage segmentation networks training:

The networks were trained using Adam optimizer with a learning rate of $1x10^{-4}$ using TensorFlow in a Titan 1080 Ti 12GB GPU or V100 32GB GPU. All the weights for the convolutional layers were randomly initialized with a Xavier uniform distribution. The training was performed with an early stopping patience criterion of 30 epochs, when validation loss non-improvement over the best validation loss was reached. Training volumes were augmented offline using a random

combination of geometric and intensity-based transforms, chosen to simulate 3D variations in patient positioning, bone shape, cartilage thickness, and MR imaging artifacts. Pooled training/validation data totaled 2812 3D-DESS volumes: 148 original volumes plus 2664 augmented volumes. Volumes were also flipped to medial-first orientation, center-cropped to 344x344x140 and normalized to their 85-th intensity percentile. 2D models were trained using slices of the original dataset, while 3D models were trained using the augmented dataset to prevent overfitting.

The cartilage and menisci segmentation dataset consisted of 176 3D-DESS volumes that were provided by IMorphics. The age and BMI for the training-validation split with the respective standard deviation was 59.9 ± 1.6 and 30.9 ± 0.7 respectively. The age and BMI for the test split with the respective standard deviation was 71.4 ± 2.9 and 30.8 ± 1.6 respectively. The sex split for training-validation and test splits was 72 males/76 females and 18 males/10 females respectively. Each of the six segmentation models was trained on an independent data split of 50 training and 98 validation volumes, with the same 28 testing volumes, for which the manual segmentation was available.

Cartilage segmentation inference and ensembling:

The 6 trained V-Nets for cartilage and menisci segmentation, were used to segment the femoral, tibial, and patellar cartilage, as well as the menisci, from a total of 47,078 3D-DESS volumes in the OAI. The inference was performed in 8 batches of 6,000 volumes and each batch lasted 3 hours. Softmax prediction values from the 3D and 2D models from each of the independent splits were ensembled to produce the final probability maps. Since the OAI only collected matching T_2 MSME, needed for the compositional T_2 spherical maps, MRI scans for the right knee of each patient, a subset of 21,118 out of the 47,078 segmented volumes were selected for this study.

OA DIAGNOSIS:

OA diagnosis model dataset:

The 21,118 spherical images were used to train a model to diagnose OA. The dataset was divided into 12,634 training, 2,558 validation and 5,926 test images, with no patient overlap across splits. The healthy controls were patient scans that had no radiographic OA (KL<2) while the positive cases were patient scans with radiographic OA (KL>1). Right knee scans for each patient were randomly assigned to a single split while controlling for the demographic factors (age, BMI, sex). To test the independence of demographic factors for the OA cases across splits, two different statistical tests were performed. The independence of sex was tested with a Pearson's chi-squared test implemented in scikit-learn[8] using Python (Python Software Foundation, https://www.python.org/). The independence of age and BMI was tested with a one-way MANOVA using a MATLAB implementation. **Table 1** summarizes the training, validation and test set splits for the bone segmentation and OA diagnosis models, along with the p-values of the statistical tests showing independence of demographic factors.

OA diagnosis network implementation:

A total of 18 binary classification models, one for each biomarker strategy per bone, were trained to extract biomarker features from the spherical biomarker representations and use them to diagnose OA (**Supplementary Figure S1d**). A Resnet[9] architecture with 50 layers (Resnet50) pre-trained with ImageNet weights was implemented in PyTorch[10]. The choice of architecture and

hyperparameters was informed by our previous study on the relationship between bone shape and radiographic OA[11]. The Resnet50 network architecture uses shortcut residual connections that improve the training performance for deeper models over similar shallower models. The basic structure of the Resnet50 follows the pattern of three convolutional layers with a 1 x 1, 3 x 3, and a 1 x 1 convolutional filter size respectively. Each of these layers is paired with batch normalization and a ReLU activation function.

All OA diagnosis model variants were initialized with ImageNet weights and fine-tuned using Adam optimizer with a learning rate of 1e-5 with a regularization weight decay value of 0.9, in order to finetune while preventing overfitting on the training set. The training was performed for 100 epochs with an early stopping 15-epoch patience for validation loss non-improvement over the best validation loss reached. The models were also trained end-to-end using a weighted binary cross entropy loss, based on the class imbalance, with a batch size of 300 in a Tesla V100 32GB GPU.

The OA diagnosis models were trained using the different biomarker strategies outlined in **Fig. 3**. The OA diagnosis models for each biomarker strategy were ensembled across the bones by averaging the softmax values outputted by each network. Therefore, each of the six biomarker models had a total of five predictive values: for the patella, for the tibia, for the femur, for the averaged predictive values of the tibia and femur, and for the average predictive values of all three bones. For the averaged ensembles, each anatomical region contributes equally to the final prediction.

SUPPLEMENTAL FIGURES

Supplementary Figure S1: (a) A bone and a cartilage segmentation model ensemble were trained on 72 and 148 manually segmented 3D-DESS volumes to segment the femur, tibia, and patella bones and corresponding cartilage. The trained models were used to segment 21,118 3D-DESS volumes. (b) Bone shape feature and cartilage thickness maps were obtained from the segmented masks. T2 values were calculated by registering 3D-DESS cartilage masks to the matching MSME MRI volumes and performing parametric T2 fitting on the cartilage. Each biomarker was then projected onto the articular bone surface, where each point contained information from each biomarker. (c) The articular bone surface projections were transformed into spherical coordinates. Six different strategies were performed to merge spherical maps per bone. (d) A total of 21,118 merged spherical maps with corresponding KL grades were used to train classifier models to diagnose radiographic OA using the biomarker learned features. A different model was trained and tested for each biomarker strategy per bone, for a total of 18 OA diagnosis models. Each of the two inputs into the OA diagnosis models represents a class in the binary classifier (healthy KL<2 vs. OA KL>1). (e) A total of 7,437 merged spherical maps with corresponding chronic pain labels were used to train classifier models pretrained on its corresponding OA diagnosis model to predict chronic pain. A different model trained and tested for each biomarker strategy per bone, for a total of 18 OA diagnosis models. Each of the two inputs into the chronic pain models represents a class in the binary classifier (chronic pain vs. no chronic pain).

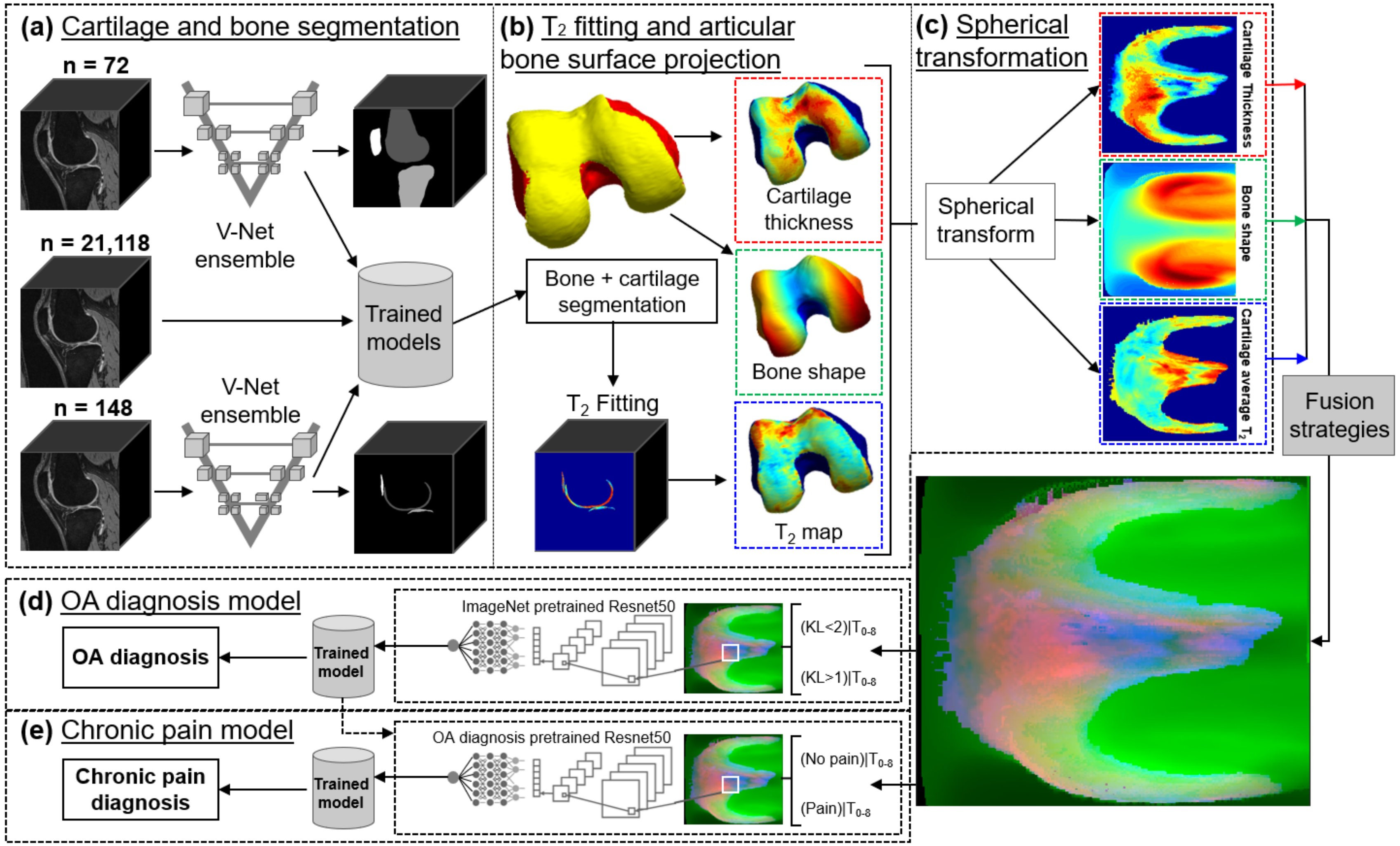

(a) Cartilage and bone segmentation
n = 72
n = 21,118
n = 148
V-Net ensemble
V-Net ensemble
Trained models
(b) T2 fitting and articular bone surface projection
Bone + cartilage segmentation
T2 Fitting
Cartilage thickness
Bone shape
T2 map
(c) Spherical transformation
Spherical transform
Cartilage Thickness
Bone shape
Cartilage average T2
Fusion strategies
(d) OA diagnosis model
OA diagnosis
Trained model
ImageNet pretrained Resnet50
(KL<2)|T0-8
(KL>1)|T0-8
(e) Chronic pain model
Chronic pain diagnosis
Trained model
OA diagnosis pretrained Resnet50
(No pain)|T0-8
(Pain)|T0-8

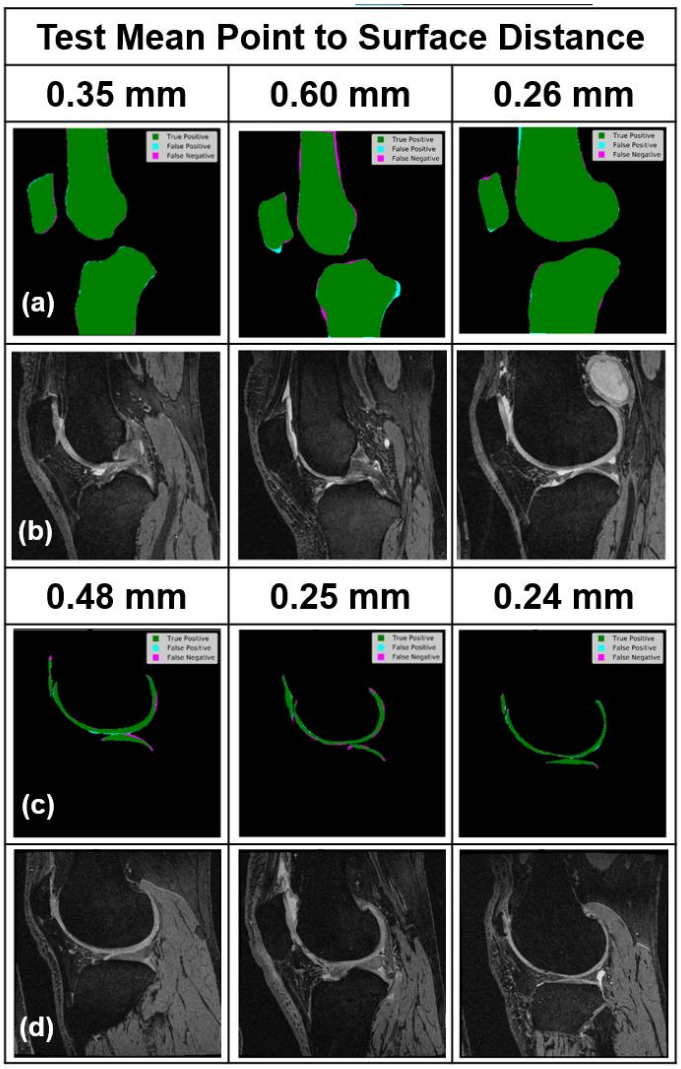

Supplementary Figure S2: Examples of bone and cartilage segmentation errors for three patients from the respective bone and cartilage segmentation test sets. Representative slices of the 3D bone and cartilage segmentation are shown along with their respective 3D-DESS images with the mean MPTS distance errors over the entire volume. The pixels in agreement between the trained segmentation model inference and the ground truths are labeled as green, representing the true positive cases. The two types of model error, false positives, where the segmentation misclassified non-bone or non-cartilage regions and false negatives, where the model missed the existing bone or cartilage, are highlighted as cyan and magenta respectively. (a, b) Bone segmentations and corresponding 3D-DESS slices for the three patients show minor errors along the articular bone surface for all three bones. The errors present can be observed along the femoral and tibial shaft, as well as the distal facet of the patella. (c, d) Cartilage segmentations and corresponding 3D-DESS slices shown for three different patients shows diffuse segmentation errors along the cartilage. Both of these errors are likely caused by signal heterogeneity and partial voluming effects. Only the articular bone surface was sampled during the spherical transformation, reducing the effect of certain bone segmentation errors along the shaft and intercondylar notch on the overall results.

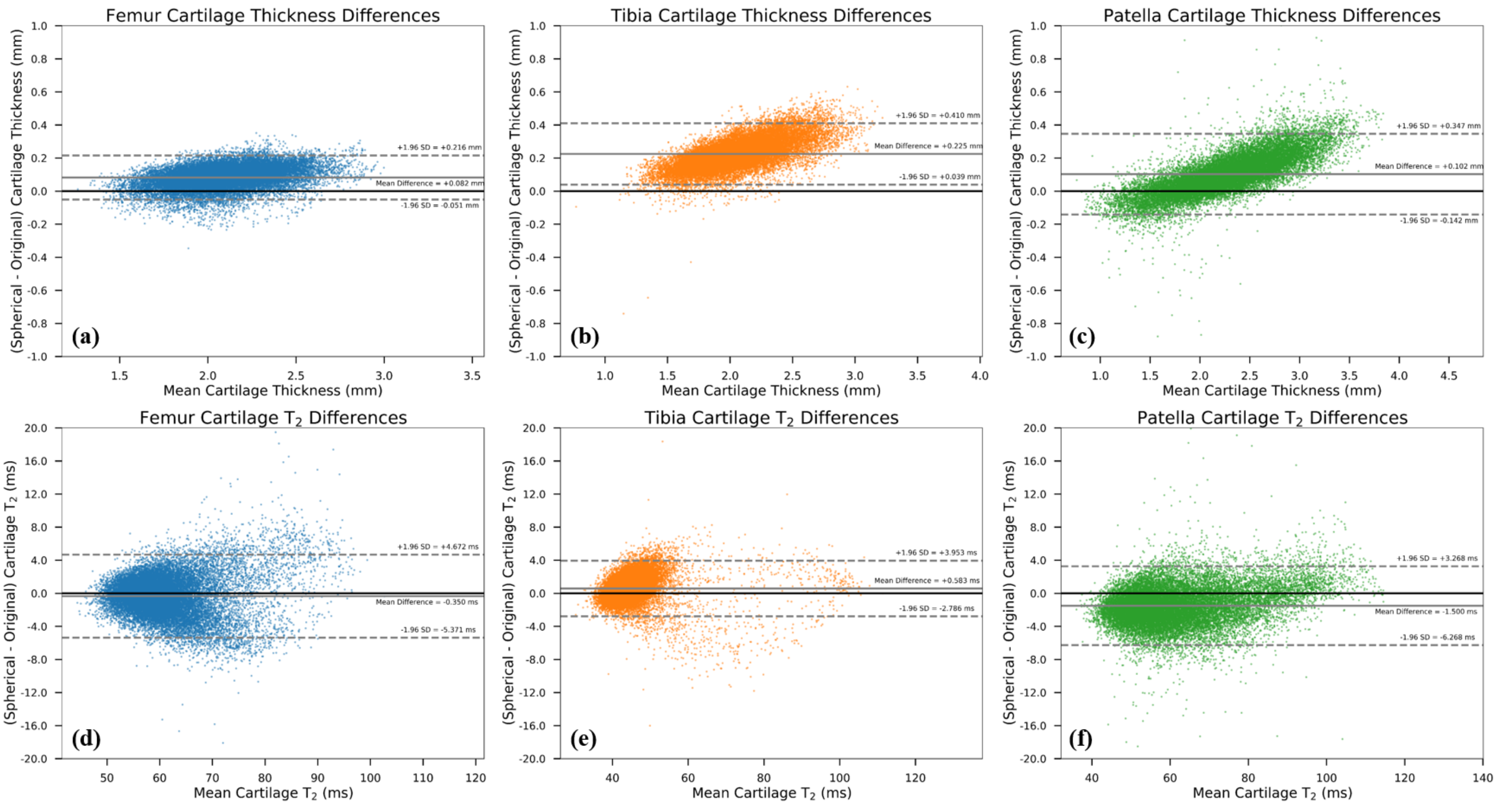

Supplementary Figure S3: Bland-Altman plots comparing the original average values of cartilage thickness and cartilage T_2 to the spherically transformed average values for each bone. The differences between the average biomarker values were calculated using the original average values as a reference, by subtracting the original average values from the average spherical values for each biomarker. The solid black line represents the zero difference. The solid gray line represents the mean difference and the dashed gray lines represent two standard deviations above or below the mean. (a) Differences between average spherical cartilage thickness and average original cartilage thickness for the femur. (b) Differences between average spherical cartilage thickness and average original cartilage thickness for the tibia. (c) Differences between average spherical cartilage thickness and average original cartilage thickness for the patella. (d) Differences between average cartilage T_2 values and average original cartilage T_2 values for the femur. (e) Differences between average cartilage T_2 values and average original cartilage T_2 values for the tibia. (f) Differences between average cartilage T_2 values and average original cartilage T_2 values for the patella.

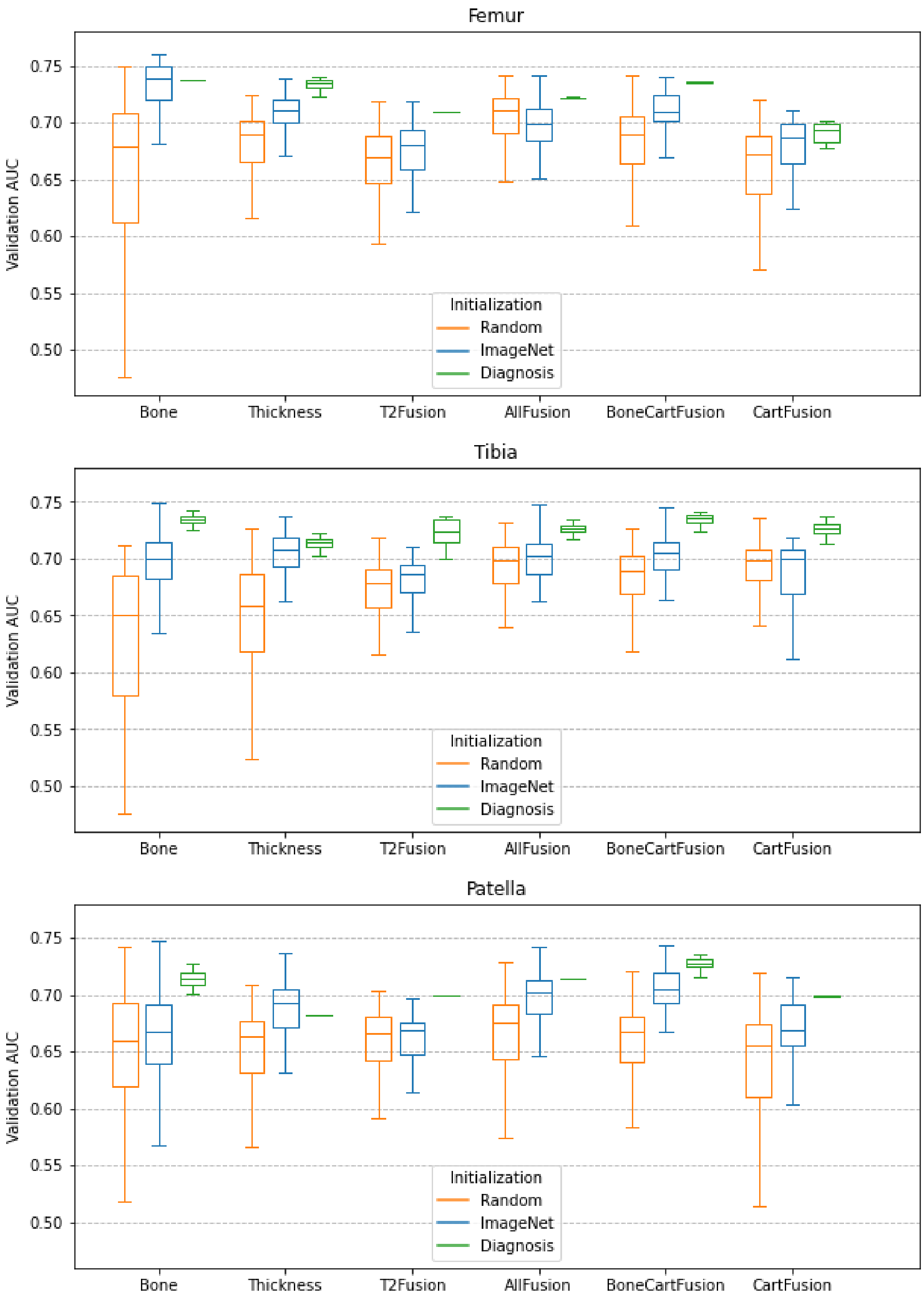

Supplementary Figure S4: Model training optimization results shown for all 18 models using the training and validation splits with two different learning rates (1x10-4 and 1x10-5), three types of Resnet (Resnet18, Resnet34, Resnet50), three initialization strategies (Random, ImageNet, OA), and four variants of layer freezing during training (first layer, first two layers, all layers, no layers), for a total of 612 combinations. The best performing models for each initialization strategy are shown with the validation AUC for each biomarker and bone.

SUPPLEMENTAL TABLES

Supplementary Table S1: Summary of the bone and cartilage segmentation test set performances, shown both as DSC and MPTS distance errors, with their corresponding 95% confidence intervals.

Segmentation model	Class	DSC (95% CI)	MPTS (mm) (95% CI)
Bone (n = 30)	Femur	98.0% (98.3, 97.7)	0.406 (0.457, 0.355)
	Tibia	98.0% (98.3, 97.7)	0.390 (0.437, 0.343)
	Patella	96.4% (97.1, 95.7)	0.370 (0.425, 0.315)
Cartilage (n = 28)	Femoral	90.0% (90.7, 89.3)	0.247 (0.268, 0.226)
	Tibial	88.6% (89.9, 86.7)	0.223 (0.259, 0.187)
	Patellar	85.7% (88.2, 82.5)	0.555 (0.749, 0.361)

Supplementary Table S2: Bootstrapped (n=100) test set OA diagnosis ROC performance for all six biomarker models per bone, as well as an average ensemble across all bones. Sensitivity, specificity, and AUC values are shown respectively, along with their corresponding 95% confidence intervals. The best performances per bone and ensemble are bolded. PTF = Patella + Tibia + Femur ensemble.

Biomarker type	Biomarker model	Test set ROC (Sensitivity/Specificity/AUC) (95% CI)			
		Patella	Tibia	Femur	PTF
Single	Cartilage T_2	67.5 (67.3, 67.7)<br>73.9 (73.7, 74.1)<br>77.6 (77.5, 77.8)	70.0 (69.8, 70.2)<br>85.3 (85.2, 85.4)<br>86.0 (85.9, 86.1)	75.5 (75.3, 75.6)<br>81.5 (81.3, 81.6)<br>86.0 (85.9, 86.1)	77.2 (77.0, 77.3)<br>87.5 (87.4, 87.6)<br>89.9 (89.8, 90.0)
	Cartilage Thickness	68.1 (67.9, 68.3)<br>72.7 (72.6, 72.9)<br>77.0 (76.9, 77.1)	68.5 (68.3, 68.7)<br>86.7 (86.6, 86.8)<br>85.5 (85.4, 85.6)	69.4 (69.2, 69.6)<br>90.9 (90.8, 91.0)<br>89.0 (88.9, 89.1)	73.7 (73.5, 73.9)<br>90.8 (90.7, 90.9)<br>90.6 (90.5, 90.7)
	Bone shape	62.2 (62.0, 62.4)<br>81.2 (81.0, 81.3)<br>78.3 (78.1, 78.4)	67.0 (66.8, 67.1)<br>91.6 (91.5, 91.7)<br>87.9 (87.8, 88.0)	73.1 (73.0, 73.3)<br>86.3 (86.2, 86.4)<br>88.5 (88.4, 88.6)	71.2 (71.0, 71.3)<br>91.9 (91.8, 92.0)<br>89.9 (89.8, 89.9)
Fusion	Morphological bone and cartilage fusion	55.3 (55.2, 55.5)<br>88.0 (87.9, 88.1)<br>80.8 (80.7, 80.9)	71.7 (71.6, 71.9)<br>89.6 (89.5, 89.7)<br>89.6 (89.5, 89.7)	**72.5 (72.3, 72.7)**<br>**90.0 (89.9, 90.1)**<br>**90.1 (90.0, 90.2)**	72.9 (72.8, 73.1)<br>93.1 (93.0, 93.2)<br>91.7 (91.6, 91.8)
	Morphological and compositional cartilage fusion	67.0 (66.8, 67.1)<br>76.7 (76.5, 76.8)<br>78.5 (78.4, 78.6)	78.0 (77.9, 78.2)<br>76.8 (76.6, 76.9)<br>86.1 (86.0, 86.2)	75.0 (74.8, 75.2)<br>83.6 (83.4, 83.7)<br>87.7 (87.6, 87.8)	78.6 (78.4, 78.7)<br>85.4 (85.3, 85.5)<br>89.5 (89.4, 89.6)
	All biomarkers fusion	**64.3 (64.2, 4.5)**<br>**83.0 (82.9, 3.1)**<br>**81.0 (80.9, 1.1)**	**76.4 (76.2, 6.5)**<br>**86.0 (85.9, 6.1)**<br>**89.8 (89.7, 9.8)**	76.3 (76.1, 6.4)<br>85.5 (85.3, 5.6)<br>89.2 (89.2, 9.3)	**78.2 (78.0, 78.3)**<br>**89.6 (89.5, 89.7)**<br>**91.7 (91.6, 91.8)**

Supplementary Table S3: Bootstrapped (n=100) test set chronic pain ROC performance for all six biomarker models per bone, as well as an average ensemble across all bones. Sensitivity, specificity, and AUC values are shown respectively, along with their corresponding 95% confidence intervals. The best performances per bone and ensemble are bolded. PTF = Patella + Tibia + Femur ensemble. Result metrics are for the last timepoint for each patient.

**Biomarker type**	**Biomarker model**	**Test set ROC (sensitivity/specificity/AUC) (95% CI)**			
		**Patella**	**Tibia**	**Femur**	**PTF**
Single	Cartilage T_2	61.0 (60.6, 61.5)<br>63.6 (63.1, 64.1)<br>67.4 (67.0, 67.7)	53.0 (52.4, 53.6)<br>77.2 (76.8, 77.6)<br>70.6 (70.2, 71.0)	64.7 (64.2, 65.2)<br>63.5 (63.1, 63.8)<br>70.0 (69.6, 70.3)	64.6 (64.1, 65.2)<br>70.7 (70.3, 71.1)<br>72.4 (72.1, 72.8)
	Cartilage thickness	60.0 (59.5, 60.5)<br>64.8 (64.3, 65.2)<br>66.2 (65.9, 66.6)	53.2 (52.7, 53.8)<br>76.8 (76.4, 77.2)<br>68.8 (68.4, 69.2)	58.2 (57.7, 58.7)<br>72.3 (71.9, 72.7)<br>70.7 (70.3, 71.0)	59.0 (58.5, 59.5)<br>72.6 (72.3, 73.0)<br>71.1 (70.7, 71.5)
	Bone shape	59.7 (59.2, 60.1)<br>72.1 (71.7, 72.6)<br>69.3 (69.0, 69.7)	56.9 (56.4, 57.4)<br>78.2 (77.8, 78.5)<br>71.1 (70.7, 71.5)	**60.2 (59.8, 60.7)**<br>**75.2 (74.8, 75.5)**<br>**72.9 (72.6, 73.3)**	59.8 (59.2, 60.3)<br>77.7 (77.3, 78.1)<br>73.4 (73.1, 73.8)
Fusion	Morphological bone and cartilage fusion	**66.2 (65.7, 66.6)**<br>**65.6 (65.2, 66.0)**<br>**71.7 (71.4, 72.0)**	**57.2 (56.7, 57.8)**<br>**79.2 (78.8, 79.6)**<br>**72.0 (71.6, 72.4)**	52.9 (52.4, 53.5)<br>76.4 (76.0, 76.8)<br>69.7 (69.4, 70.0)	**57.5 (57.0, 58.0)**<br>**78.1 (77.7, 78.4)**<br>**73.2 (72.9, 73.6)**
	Morphological and compositional cartilage fusion	59.3 (58.7, 59.9)<br>61.3 (60.9, 61.7)<br>67.1 (66.7, 67.4)	51.2 (50.6, 51.7)<br>76.7 (76.4, 77.1)<br>71.2 (70.8, 71.5)	53.2 (52.6, 53.7)<br>80.5 (80.2, 80.8)<br>72.9 (72.6, 73.3)	57.9 (57.4, 58.4)<br>76.4 (76.1, 76.7)<br>73.1 (72.7, 73.4)
	All biomarkers fusion	55.1 (54.5, 55.6)<br>74.0 (73.6, 74.4)<br>69.5 (69.2, 69.9)	51.7 (51.2, 52.2)<br>80.0 (79.6, 80.3)<br>72.8 (72.4, 73.1)	57.1 (56.6, 57.6)<br>76.8 (76.4, 77.2)<br>70.9 (70.6, 71.3)	54.2 (53.7, 54.7)<br>80.8 (80.5, 81.2)<br>73.1 (72.7, 73.4)

REFERENCES:

[1] Milletari, F., Navab, N. & Ahmadi, S.-A. V-Net: Fully Convolutional Neural Networks for Volumetric Medical Image Segmentation. (2016).

[2] Drozdzal, M., Vorontsov, E., Chartrand, G., Kadoury, S. & Pal, C. The Importance of Skip Connections in Biomedical Image Segmentation. *ArXiv160804117 Cs* (2016).

[3] Caliva, F., Iriondo, C., Martinez, A. M., Majumdar, S. & Pedoia, V. Distance Map Loss Penalty Term for Semantic Segmentation. *ArXiv190803679 Cs Eess* (2019).

[4] Pereyra, G., Tucker, G., Chorowski, J., Kaiser, Ł. & Hinton, G. Regularizing Neural Networks by Penalizing Confident Output Distributions. *ArXiv170106548 Cs* (2017).

[5] Kingma, D. P. & Ba, J. Adam: A Method for Stochastic Optimization. *ArXiv14126980 Cs* (2014).

[6] Glorot, X. & Bengio, Y. Understanding the difficulty of training deep feedforward neural networks. 8.

[7] Iriondo, C. *et al.* Towards Understanding Mechanistic Subgroups of Osteoarthritis: 8 Year Cartilage Thickness Trajectory Analysis. *J. Orthop. Res.* **n/a**,.

[8] Pedregosa, F. *et al.* Scikit-learn: Machine Learning in Python. *Mach. Learn. PYTHON* 6.
[9] He, K., Zhang, X., Ren, S. & Sun, J. Deep Residual Learning for Image Recognition. *ArXiv151203385 Cs* (2015).
[10] Paszke, A. *et al.* Automatic differentiation in PyTorch. in 4.
[11] Martinez, A. M. *et al.* Learning osteoarthritis imaging biomarkers from bone surface spherical encoding. *Magn. Reson. Med.* **n/a**,.
